# Supplementary figures and images for: Personality reflection in the brain’s intrinsic functional architecture remains elusive
Source: PLoS One. 2020 Jun 2;15(6):e0232570. doi: 10.1371/journal.pone.0232570 (PMC7266317; doi:10.1371/journal.pone.0232570)

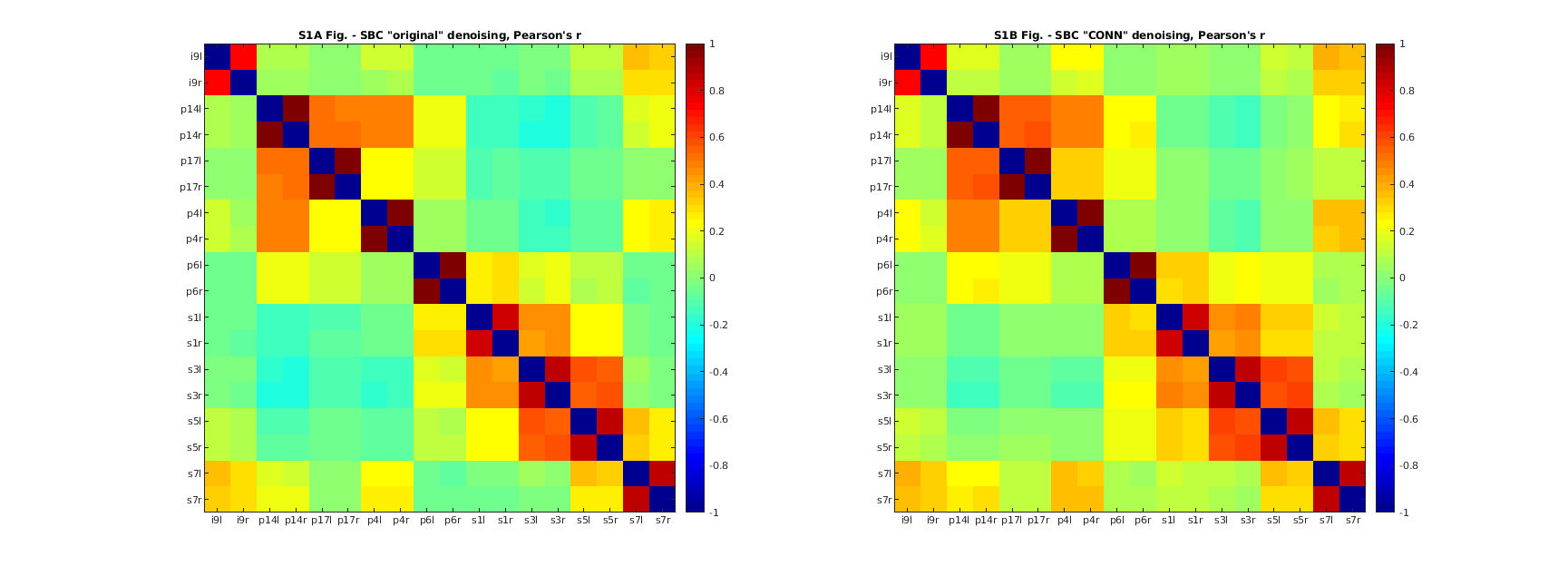

Supplement: S1 Fig — Seed-based connectivity between 18 ROIs using the original (S1A Fig) and the default CONN denoising (S1B Fig). (TIF) [file pone.0232570.s001.tif]

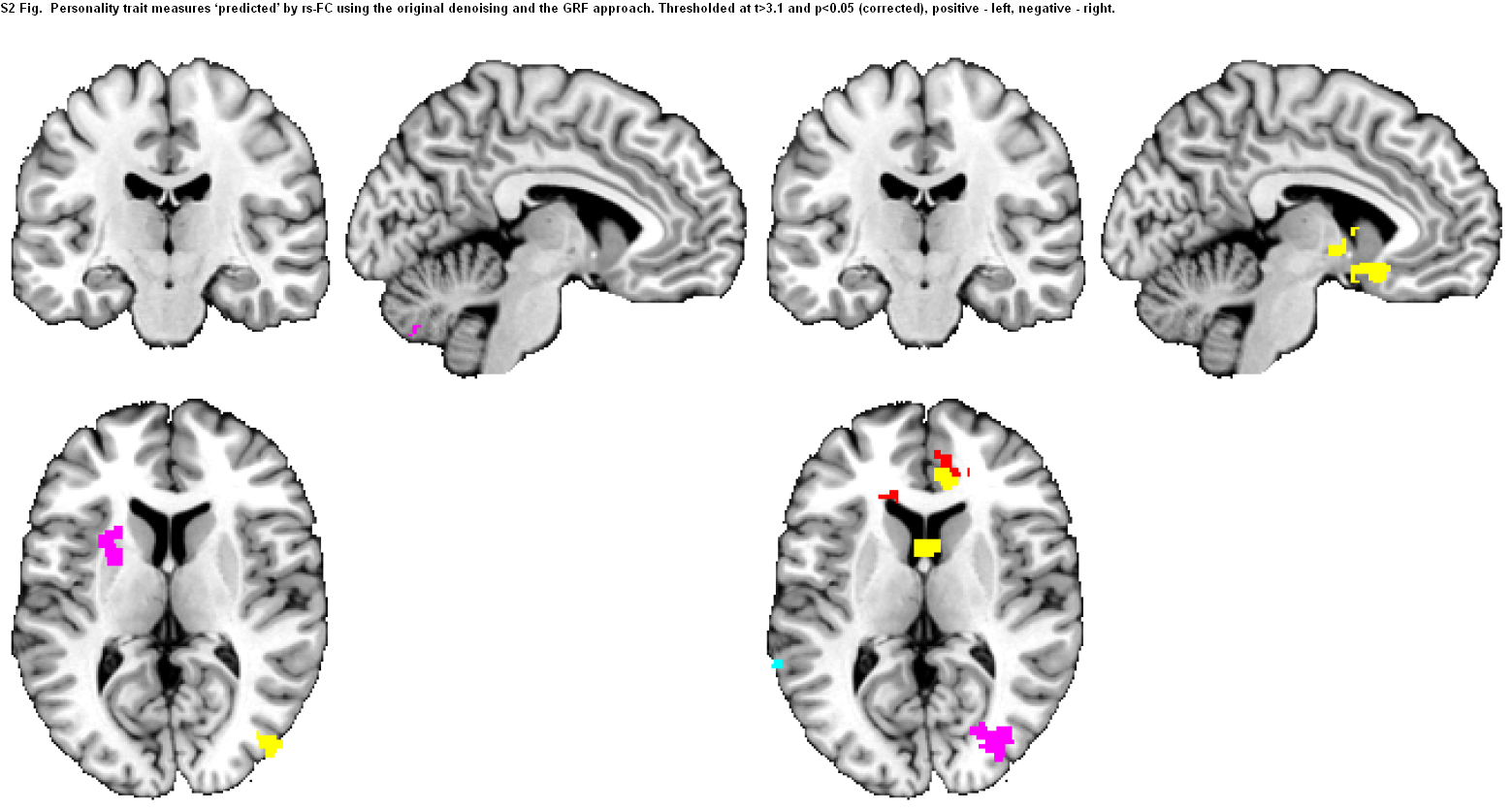

Supplement: S2 Fig — Thresholded at t>3.1 and p<0.05 (corrected), positive—left, negative—right. (TIF) [file pone.0232570.s002.tif]

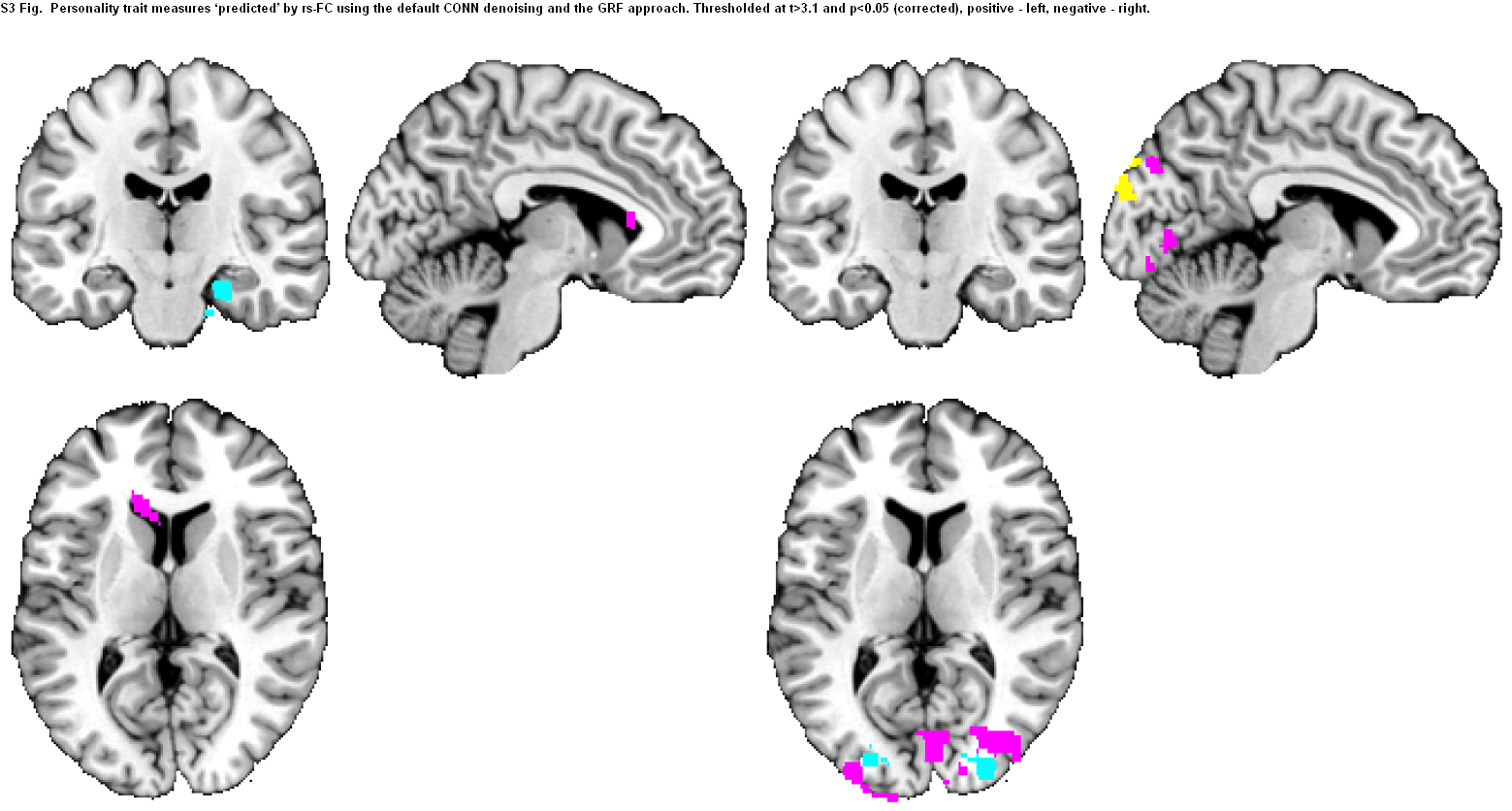

Supplement: S3 Fig — Thresholded at t>3.1 and p<0.05 (corrected), positive—left, negative—right. (TIF) [file pone.0232570.s003.tif]
