# Supplementary material for: Personality reflection in the brain’s intrinsic functional architecture remains elusive
Source: PLoS One. 2020 Jun 2;15(6):e0232570. doi: 10.1371/journal.pone.0232570 (PMC7266317; doi:10.1371/journal.pone.0232570)
Supplement: S1 Table — (PDF) [file pone.0232570.s004.pdf]

S1 Table. MNI coordinates of seed ROIs.

	x	y	z
I9l	-5	25	-10
I9r	5	25	-10
P4l	-2	-36	35
P4r	2	-36	35
P6l	-2	-47	58
P6r	2	-47	58
P14l	-2	-64	45
P14r	2	-64	45
P17l	-1	-78	43
P17r	1	-78	43
S1l	-5	-10	47
S1r	5	-10	47
S3l	-5	14	42
S3r	5	14	42
S5l	-5	24	28
S5r	5	24	28
S7l	-5	47	11
S7r	5	47	11
