# Supplementary material for: Personality reflection in the brain’s intrinsic functional architecture remains elusive
Source: PLoS One. 2020 Jun 2;15(6):e0232570. doi: 10.1371/journal.pone.0232570 (PMC7266317; doi:10.1371/journal.pone.0232570)
Supplement: S2 Table — (PDF) [file pone.0232570.s005.pdf]

S2 Table. Smoothness estimates for the first-level statistical maps

ADELSTEIN		CONN	
<<< n >>>			
i9l	0.03368	i9l	0.04984
i9r	0.03265	i9r	0.04948
p14l	0.04109	p14l	0.04857
p14r	0.03981	p14r	0.04679
p17l	0.03697	p17l	0.04600
p17r	0.03739	p17r	0.04546
p4l	0.04969	p4l	0.06114
p4r	0.04908	p4r	0.05973
p6l	0.03903	p6l	0.05601
p6r	0.03664	p6r	0.05486
s1l	0.03848	s1l	0.05239
s1r	0.03980	s1r	0.05772
s3l	0.04162	s3l	0.03788
s3r	0.03848	s3r	0.04170
s5l	0.04091	s5l	0.04467
s5r	0.04615	s5r	0.04620
s7l	0.04034	s7l	0.05261
s7r	0.03706	s7r	0.05295
<<< e >>>			
i9l	0.03508	i9l	0.04991
i9r	0.03439	i9r	0.05576
p14l	0.03591	p14l	0.03883
p14r	0.03887	p14r	0.04150
p17l	0.04445	p17l	0.04581
p17r	0.04310	p17r	0.04477
p4l	0.03841	p4l	0.04367
p4r	0.03796	p4r	0.04391
p6l	0.03589	p6l	0.04867

p6r	0.03813	p6r	0.04849
s1l	0.03889	s1l	0.04908
s1r	0.04343	s1r	0.05004
s3l	0.03304	s3l	0.03767
s3r	0.03289	s3r	0.04210
s5l	0.03573	s5l	0.04980
s5r	0.03195	s5r	0.04777
s7l	0.03894	s7l	0.04877
s7r	0.03448	s7r	0.04592
<<< o >>>			
i9l	0.04540	i9l	0.04986
i9r	0.03758	i9r	0.04971
p14l	0.03761	p14l	0.04416
p14r	0.03942	p14r	0.04284
p17l	0.03092	p17l	0.03265
p17r	0.03209	p17r	0.03535
p4l	0.03339	p4l	0.04086
p4r	0.03396	p4r	0.03991
p6l	0.03823	p6l	0.03319
p6r	0.03812	p6r	0.03122
s1l	0.03709	s1l	0.04975
s1r	0.03894	s1r	0.05275
s3l	0.03351	s3l	0.04477
s3r	0.03748	s3r	0.04790
s5l	0.02935	s5l	0.04606
s5r	0.03235	s5r	0.04537
s7l	0.03269	s7l	0.03704
s7r	0.03435	s7r	0.04093
<<< a >>>			
i9l	0.04358	i9l	0.05131
i9r	0.04022	i9r	0.05315
p14l	0.03864	p14l	0.03691
p14r	0.03793	p14r	0.03612

p17l	0.03004	p17l	0.03945
p17r	0.02900	p17r	0.03936
p4l	0.03567	p4l	0.03818
p4r	0.03483	p4r	0.03897
p6l	0.03675	p6l	0.03878
p6r	0.03745	p6r	0.03994
s1l	0.04345	s1l	0.05132
s1r	0.04964	s1r	0.05375
s3l	0.03497	s3l	0.03834
s3r	0.03485	s3r	0.04309
s5l	0.03358	s5l	0.03820
s5r	0.03310	s5r	0.02980
s7l	0.03360	s7l	0.04085
s7r	0.02798	s7r	0.03978
<<< c >>>			
i9l	0.04339	i9l	0.05836
i9r	0.03204	i9r	0.04717
p14l	0.04193	p14l	0.05428
p14r	0.04104	p14r	0.05534
p17l	0.03158	p17l	0.02555
p17r	0.02933	p17r	0.02318
p4l	0.02292	p4l	0.04167
p4r	0.02346	p4r	0.04096
p6l	0.02858	p6l	0.03188
p6r	0.03127	p6r	0.03353
s1l	0.02278	s1l	0.03804
s1r	0.02326	s1r	0.03918
s3l	0.03052	s3l	0.03407
s3r	0.03574	s3r	0.04041
s5l	0.03645	s5l	0.04133
s5r	0.03591	s5r	0.04515
s7l	0.02978	s7l	0.04374
s7r	0.02343	s7r	0.04458
