# Supplementary material for: Personality reflection in the brain’s intrinsic functional architecture remains elusive
Source: PLoS One. 2020 Jun 2;15(6):e0232570. doi: 10.1371/journal.pone.0232570 (PMC7266317; doi:10.1371/journal.pone.0232570)
Supplement: S4 Table — (PDF) [file pone.0232570.s007.pdf]

S4 Table. A number of analyses with statistically significant results when using the default CONN denoising and the GRF approach, thresholded at t>3.1, p<0.05 (corrected).

analysis	n	e	o	a	c	total
positive	2	2	2	3	0	9
negative	2	0	0	5	7	14
total	4	2	2	8	7	23
